# Association of genetic variants, protein domains, and phenotypes in the ZMIZ1 syndromic neurodevelopmental disorder

**DOI:** 10.3389/fnins.2025.1605762

**Published:** 2025-06-03

**Authors:** Kendall E. Cormier, Belle L. Kantor, Rajan K C, Xabier Blanco-Fernandez, Maria J. Galazo

**Affiliations:** ^1^Department of Cell and Molecular Biology, Tulane University, New Orleans, LA, United States; ^2^Picower Institute for Learning and Memory, Massachusetts Institute of Technology, Cambridge, MA, United States; ^3^Tulane Brain Institute, Tulane University, New Orleans, LA, United States

**Keywords:** ZMIZ1, autism, intellectual disability, alanine-rich domain, TPR-domain

## Abstract

**Background:**

Human genetic studies have linked loss-of-function variants in Zinc Finger MIZ-Type Containing 1 (ZMIZ1) to a spectrum of neurodevelopmental disorders (NDDs), such as intellectual disability (ID), autism spectrum disorders (ASD), and attention-deficit/hyperactivity disorder (ADHD). Recently, multiple studies have reported ZMIZ1 variants in patients with NDDs, in some cases providing detailed phenotypic descriptions of the carriers. However, how ZMIZ1 variants may contribute to the phenotypic variability of carriers and the different phenotypic manifestations of NDDs has not been explored.

**Methods:**

Here, we examine the relationship between ZMIZ1 variants, affected ZMIZ1 protein domains, and phenotypic variability of individuals diagnosed with NDD using de-identified data from 15 publicly available studies describing mutations in ZMIZ1. This study includes descriptions of ZMIZ1 disease-associated variants of 36 individuals diagnosed with NDDs: 35 single-nucleotide variants (SNVs) and 1 deletion, all in the coding sequence. Pathogenicity scores and records for these variants were obtained from AlphaMissense, PolyPhen, and ClinVar and were correlated to the variants’ locations across protein domains. Phenotypic descriptions were obtained from publicly available reports. To further explore the potential functional impact of SNV on ZMIZ1, protein folding predictions of wild-type and mutated ZMIZ1 were performed using AlphaFold.

**Results:**

We find that patients with SNVs in the Alanine-rich domain show strong association with diagnosis of ID (62.5%), motor delay (70%), and other physical phenotypic manifestations (100%), while ASD diagnosis in combination with ID is more strongly associated with mutations in TPR and Proline-rich domains. Morphological alterations in the brain and cranium are highly prevalent in individuals with missense mutations in ZMIZ1, without any association to specific protein domains. Missense mutations in the Alanine-rich and TPR domains are predicted to alter the relative position of domains and ZMIZ1 3D configuration.

**Conclusion:**

Overall, our study highlights the impact of mutations across ZMIZ1 domains and their association with distinct neurodevelopmental phenotypes in individuals with ZMIZ1 variants, which will lead to better interpretation of ZMIZ1 variants and diagnosis of patients with ZMIZ1 neurodevelopmental syndrome.

## Introduction

Neurodevelopment is a highly regulated multi-step process underlying the formation and wiring of the nervous system. Disruptions in neurodevelopmental processes result in a wide variety of neurodevelopmental disorders (NDDs), leading to impaired cognition, communication, adaptive behavior, and psychomotor skills (Gidziela et al., 2023). NDDs such as autism spectrum disorder (ASD), attention deficit hyperactivity disorder (ADHD), and intellectual disability (ID), are important contributors to disability globally, collectively affecting 15% of children and adolescents worldwide and >20% of the population in the United States (Gidziela et al., 2023; Iossifov et al., 2014; Guo et al., 2019; Ruzzo et al., 2019).

NDDs are caused by a complex interplay of genetic, epigenetic, and environmental risk factors (Rajan et al., 2024; Sahin and Sur, 2015). In the past 20 years, there has been an increased appreciation of the impact of genetic variants on NDD pathogenesis, which has led to considerable progress in identifying NDD-associated genes (Myers et al., 2020; Fu et al., 2022; Warrier et al., 2022). Clinically distinct NDDs, including ASD, ID, and ADHD, have shared genetic etiologies, such as rare multigenic copy number variants (CNVs) and single-gene variants producing deleterious impacts on brain function (Shimelis et al., 2023). Loss-of-function (or disease-associated) variants affecting one gene can be associated with multiple NDDs. How variants in NDD-associated genes contribute to inter-individual phenotypic variability of carriers leading to the diagnosis of distinct NDDs, or to different phenotypic manifestations of a specific NDD is not understood (Chen et al., 2016; Szatmari, 2018).

Recent human genetic studies have linked variants in Zinc Finger MIZ-Type Containing 1 (ZMIZ1) with a spectrum of NDDs, such as ID, ASD, and ADHD (Carapito et al., 2019; Liu et al., 2018; Latchman et al., 2020; Córdova-Fletes et al., 2015; Lu et al., 2022; Phetthong et al., 2021). ZMIZ1 is a chromatin remodeler and transcriptional activator regulating important biological processes spanning embryonic development, angiogenesis, immune response, and has been associated with conditions such as cancer, leukemia, and diabetes in addition to NDDs (Beliakoff and Sun, 2006; Alghamdi et al., 2022; Rakowski et al., 2013; Takata et al., 2019; Zhang et al., 2022; Rajan et al., 2024; Jiang et al., 2020; Mathios et al., 2019; Zhou et al., 2022; Wang et al., 2018). ZMIZ1 functions in neurodevelopment have only recently begun to be investigated (Rajan et al., 2024; Rajan et al., 2024). ZMIZ1 is a transcriptional co-activator highly expressed during embryonic brain development in both mice and humans, especially in regions such as the cortex, hippocampus, and cerebellum, areas notably affected in intellectual disability (ID) and autism spectrum disorder (ASD) (Rajan et al., 2024). ZMIZ1 interacts with key neurodevelopmental pathways Notch1, androgen receptor, p53, and Smad3/4 (Beliakoff and Sun, 2006; Pinnell et al., 2015; Lee et al., 2007; Li et al., 2006), which regulate neurogenesis, differentiation, dendritic development, and subsequently neural circuitry (Kaltezioti et al., 2010; Liu et al., 2013; La Rosa et al., 2021; Liu et al., 2016; Redmond et al., 2000; Breunig et al., 2007). Moreover, ZMIZ1 interacts with proteins of the SWI/SNF-like BAF chromatin remodeling complex, crucial for neuronal differentiation, dendritic development, and synapse development (Córdova-Fletes et al., 2015; Wu et al., 2007; Deng et al., 2015; Zhang et al., 2016; Li et al., 2011). Recently, transcriptomic analysis has shown altered expression of ASD-associated genes in ZMIZ1 mutant mice (Rajan et al., 2024; Rajan et al., 2024).

ZMIZ1 belongs to the Zinc finger MIZ-type family of proteins, containing a highly conserved SPRING/MIZ domain, which is important for transcriptional regulation and confers SUMO-E3-ligase activity (Lee et al., 2007; Li et al., 2006; Rytinki et al., 2009; Schmidt and Müller, 2003; Sharma, 2003; Huang et al., 2005). ZMIZ1 also includes other important functional domains, such as an N-terminal tetratricopeptide repeat (TPR) responsible for interaction with NOTCH1 (Wang et al., 2018; Pinnell et al., 2015), and a large intrinsically disordered region, which is predicted to confer extremely dynamic behavior to proteins to facilitate the formation of multi-molecular complexes (Rajan et al., 2024; Pinnell et al., 2015). ZMIZ1 disordered region includes an Alanine-rich motif and two Proline-rich domains, one of them within the C-terminus, containing a strong transactivation domain (TAD) (Lee et al., 2007; Sharma, 2003; Huang et al., 2005).

Although variants with different pathogenic potential have been found distributed across ZMIZ1 coding sequence, it has been reported that the Alanine-rich domain accumulates the highest burden of disease-causing variants (Rajan et al., 2024), suggesting a relationship between variants, ZMIZ1 protein domains, and phenotypes. However, the potential relationship between ZMIZ1 variants, affected domains, and phenotypic variability of NDDs in affected carriers has not been studied. Here, we use publicly available resources and in silico analysis of reported ZMIZ1 single nucleotide variants (SNVs) associated with NDDs to investigate their distribution across ZMIZ1 and the potential association of disease-causing variants in distinct protein domains with specific neurodevelopmental phenotypes. Our analysis reveals a strong association between variants in the Alanine-rich domain and ID diagnosis, while ASD diagnosis in combination with ID is more strongly associated with variants in the TPR and Proline-rich domains. Most reported disease-associated variants in the Proline-Rich domains lead to frameshifts and early sequence termination. Because the Alanine-rich domain has the highest accumulation of disease-causing variants, and SNVs in the TPR domain were not previously linked to neurological phenotypes of ZMIZ1 neurodevelopmental syndrome, we performed 3D modeling and prediction analysis of SNVs reported in these domains. Prediction models suggest that even SNVs leading to changes in a single amino acid residue in these domains may affect ZMIZ1 structure, underscoring the need for further investigation of the effects of these variants.

In summary, our study highlights the importance of TPR and Alanine-rich domains for ZMIZ1 functions in the brain, their potential association with distinct neurodevelopmental phenotypes, and lays the foundation for future work on the contribution of specific ZMIZ1 variants and functional domains to the interindividual phenotypic variability, which will lead to better interpretation of ZMIZ1 variants and diagnosis of patients with ZMIZ1 neurodevelopmental syndrome.

## Results

### Predicted pathogenicity of variants across ZMIZ1 protein domains

From a collection of 15 studies (Carapito et al., 2019; Latchman et al., 2020; Córdova-Fletes et al., 2015; Lu et al., 2022; Phetthong et al., 2021; He et al., 2024; Sheth et al., 2023; Alqahtani et al., 2023; Zhou et al., 2022; Deng et al., 2022; Valind et al., 2021; Bartolomaeus et al., 2023; Brea-Fernández et al., 2022; Schluth-Bolard et al., 2019; Kruidenier et al., 2025) describing patients with mutations in ZMIZ1 and diagnosed NDDs or developmental delay, we selected for this study 36 unique *de novo* mutations (35 SNVs and 1 deletion) in the ZMIZ1 protein coding region. We did not include any cases with ZMIZ1 mutations classified as translocations, inversions, or variants in splice donors/acceptors.

**Figure 1:**
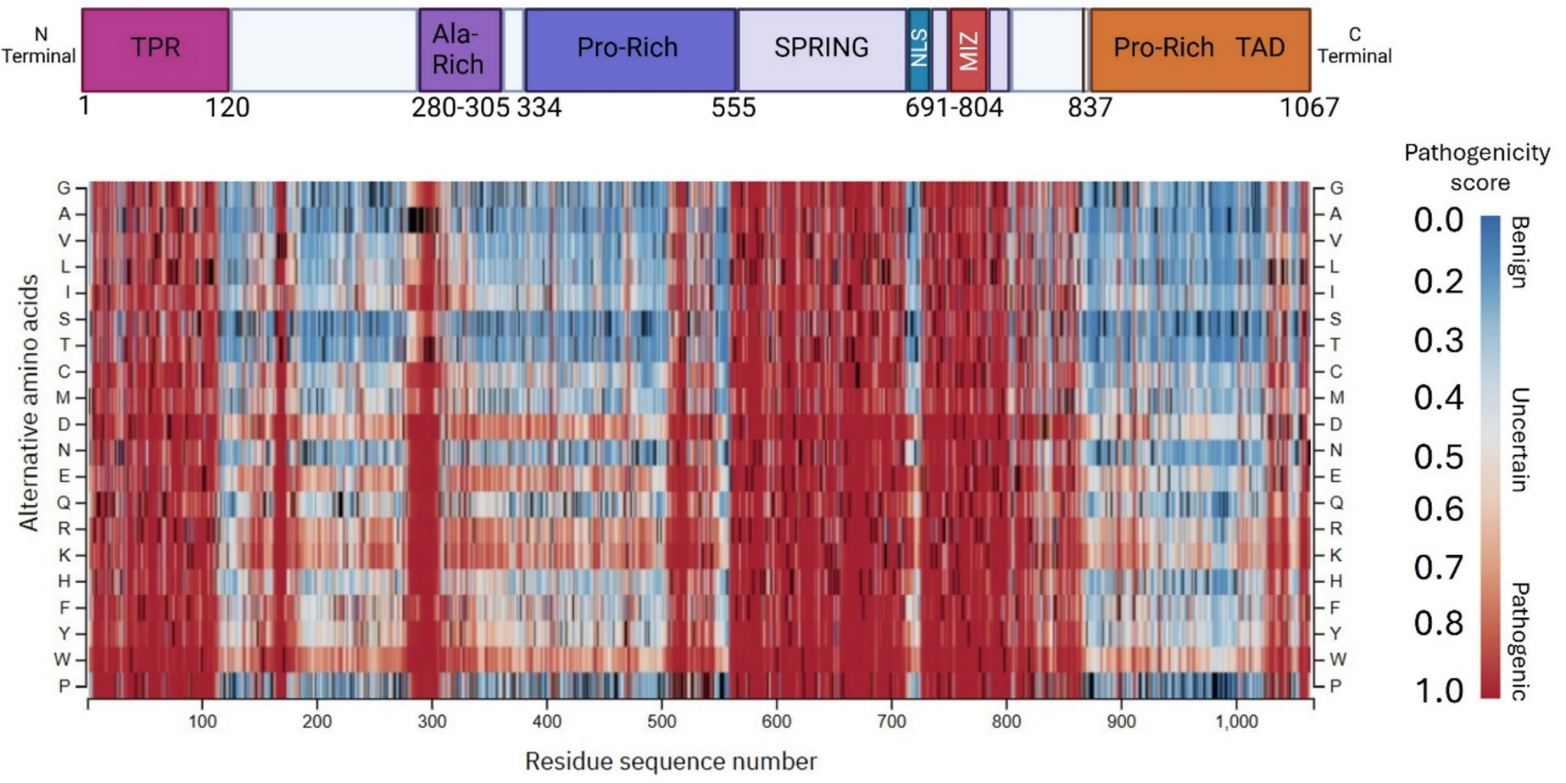
Predicted pathogenicity of single nucleotide variants across ZMIZ1 coding sequence. Top panel, schematic of ZMIZ1 and protein domains: N-terminal tetratricopeptide repeat (TPR), Alanine-rich domain (Ala-Rich), Proline-rich domain (Pro-Rich), SPRING/MIZ type zinc finger domain (SPRING/MIZ), nuclear localization signal (NLS), transactivation domain (TAD). Numbers under ZMIZ1 denote amino acid residues at the beginning and end of each labeled domain. Bottom panel, AlphaMissense pathogenicity prediction map categorizes every predicted missense mutation on each residue as either “benign,” “uncertain,” or “pathogenic” producing a score that estimates the likelihood of a variant being pathogenic (“benign” scores 0.0–0.34; “uncertain” scores 0.34–0.564; “pathogenic” scores 0.564–1.0).

*De novo* variants are distributed throughout the ZMIZ1 sequence. It has been recently reported that the Alanine-Rich domain presents the highest accumulation of disease-associated SNVs and variants of uncertain pathogenic significance (Rajan et al., 2024). To compare the frequency of reported disease-causing or uncertain-significance SNVs with the predicted pathogenic effect of mutations across ZMIZ1, we determined the predicted pathogenic effect of missense mutations for all residues in ZMIZ1 using AlphaMissense prediction model. Mutations in each residue were classified as “benign,” “uncertain” or “pathogenic” according to their predicted pathogenicity score (benign, 0–0.34; uncertain, 0.34–0.564, pathogenic, 0.564–1.0) (Figure 1).

Consistent with previous observations (Rajan et al., 2024), our analysis confirmed strong clustering of predicted pathogenic mutations (indicated as tones of red) in the Alanine-rich domain. Interestingly, our analysis also showed strong clustering of predicted pathogenic mutations in the TPR, SPRING/MIZ, C-terminal region of the central Proline-rich, and C-terminal TAD domains (Figure 1). These results suggest that mutations in these domains may also be highly associated with pathological conditions and thus, that they are essential for ZMIZ1 functions.

### Association of ZMIZ1 variants, domains, and neurodevelopmental phenotypes

**Figure 2:**
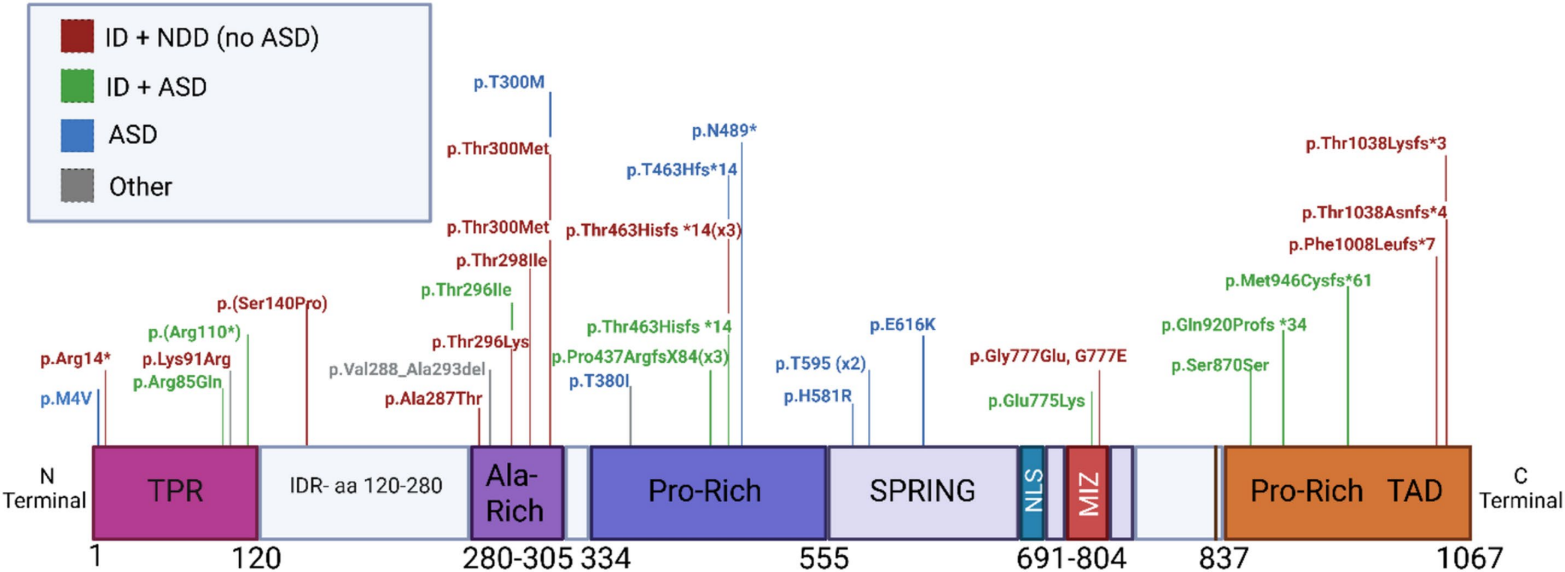
Location of *de novo* mutations in patients with NDD and other developmental disorders across ZMIZ1. Protein map illustrating the *de novo* mutations in patients with NDD and other developmental disorders. Variants are classified according to the neurodevelopmental phenotype observed in the patient. This map does not include translocations or reciprocal fusions. ID + NDD: diagnosis of intellectual disability and other neurodevelopmental conditions excluding ASD (red). ID + ASD: diagnosis of intellectual disability and ASD (green). ASD: diagnosis of ASD (blue). Other: diagnosis of developmental alterations without NDD (grey). Variants with the same phenotypic classification found in more than one individual are followed by parentheses indicating the number of individuals with the variant (e.g., X3).

To determine whether there is a putative association between mutations in ZMIZ1 coding region, the protein domain affected, and the NDD phenotype observed, we reviewed the case descriptions of 36 individuals carrying ZMIZ1 variants (35 SNVs and 1 deletion) with developmental and neurodevelopmental alterations. We classified their neurodevelopmental phenotypes and analyzed the distribution of variants across the ZMIZ1 sequence. The depth of the phenotypic description of individuals varies depending on the study. In some cases, where ZMIZ1 variants were reported through studies designed to identify new genes by next-generation sequencing in large cohorts of ID/ASD probands, clinical descriptions were limited. Since ID/ASD status was reported in most selected individuals, we classified developmental/neurodevelopmental phenotypes as follows: (1) ID (when ID is the main neurodevelopmental deficit, although it is typically presented in conjunction with other developmental abnormalities such as speech delay); (2) ID presented with ASD, (3) ASD without ID, (4) ID + NDD (when ID in addition to other neurodevelopmental disorders, other than ASD, are the main neurological phenotypes), and (5) other developmental phenotypes (typically face or hands/feet dysmorphism and motor delay). For these 36 individuals’ variants, we summarized their reported pathogenicity according to ClinVar, PolyPhen (if available), and their predicted pathogenicity according to AlphaMissence (Supplementary Table 1), as well as the described developmental/neurodevelopmental phenotype of the carriers (Figure 2).

Putative associations between domain location, mutation type, and phenotype were investigated. SNVs in the Alanine-rich domain have a greater incidence of pathogenicity in the form of an ID diagnosis. In contrast, pathogenic SNVs in the central Proline-rich and SPRING domains have a higher association with ASD diagnosis.

In agreement with previous reports (Rajan et al., 2024; Carapito et al., 2019), our analysis showed a strong association between variants in the Alanine-rich domain and NDDs (8/8 cases), specifically with ID diagnosis (62.5%; 5/8 cases). However, diagnosis of ID with ASD as comorbidity (12.5%; 1/8 cases), or ASD alone (12.5%; 1/8 cases) were not as prevalent (Figure 2). All variants described in the Alanine-rich domain in NDD patients correspond to SNVs leading to missense mutations.

Consistent with our results of predicted pathogenicity of residue mutations in the TPR, SPRING/MIZ, and Proline-rich domains (Figure 1), we found a significant association between NDD diagnosis and variants in these domains. Of the 36 variants found in carriers with NDDs, 27 (75%) were located in these domains (Figure 2).

One hundred percent of cases with variants in the TPR presented NDD diagnosis and not other developmental abnormalities. Specifically, 40% of individuals (2/5 cases) presented ID diagnosis, 40% (2/5 cases) presented ID and ASD, and 20% (1/5 cases) were reported as ASD without ID (Figure 2). Sixty percent of the TPR variants produced missense mutations and 40% produced nonsense mutations. However, in the central Proline-rich domain, 90% of variants (9/10) produced frameshifts leading to early termination of the ZMIZ1 sequence (Figure 2). Regarding the phenotypes reported for individuals carrying variants in the central Proline-rich domain, 40% of individuals (4/10 cases) presented ID and ASD, 30% (3/10) presented ID and other developmental comorbidities, and 30% of individuals (3/10) presented ASD as the only phenotype reported (Figure 2). Thus, all mutations in the central Proline-rich domain are associated with some form of NDDs with ASD diagnosis being highly prevalent.

All SPRING/MIZ variants reported correspond to SNVs producing missense amino acid substitutions. Phenotypes reported for variants affecting the SPRING/MIZ domain correlate to NDDs rather than other developmental abnormalities. Sixty-six percent of cases (4/6) correspond to individuals with ASD as the only phenotype reported, 16.6% of individuals (1/6) presented ASD and ID, and 16.6% of individuals (1/6) presented ID. This may suggest a stronger association between the presence of variants in this domain and ASD diagnosis.

In the C-terminal Proline-rich TAD domain 83.3% (5/6) of SNVs produced frameshifts leading to early termination of ZMIZ1 sequence (Figure 2). A high prevalence of variants leading to frameshifts was also observed in the central Proline-rich domain, suggesting a common trend in both Proline-rich domains for variants to produce truncated or non-functional ZMIZ1 protein. Regarding the phenotypes reported for variants affecting the C-terminal Proline-rich TAD domain, 50% of individuals (3/6) presented ASD and ID, and 50% of individuals (3/6) presented ID with other developmental abnormalities (Figure 2). Overall, these results suggest a trend in the diagnosed NDD phenotypes where patients with variants in the Alanine-rich domain are more likely to present ID, in contrast, patients with variants in the TPR, SPRING/MIZ, and Proline-rich domains are more likely to present ID in combination with ASD phenotypes.

**Figure 3:**
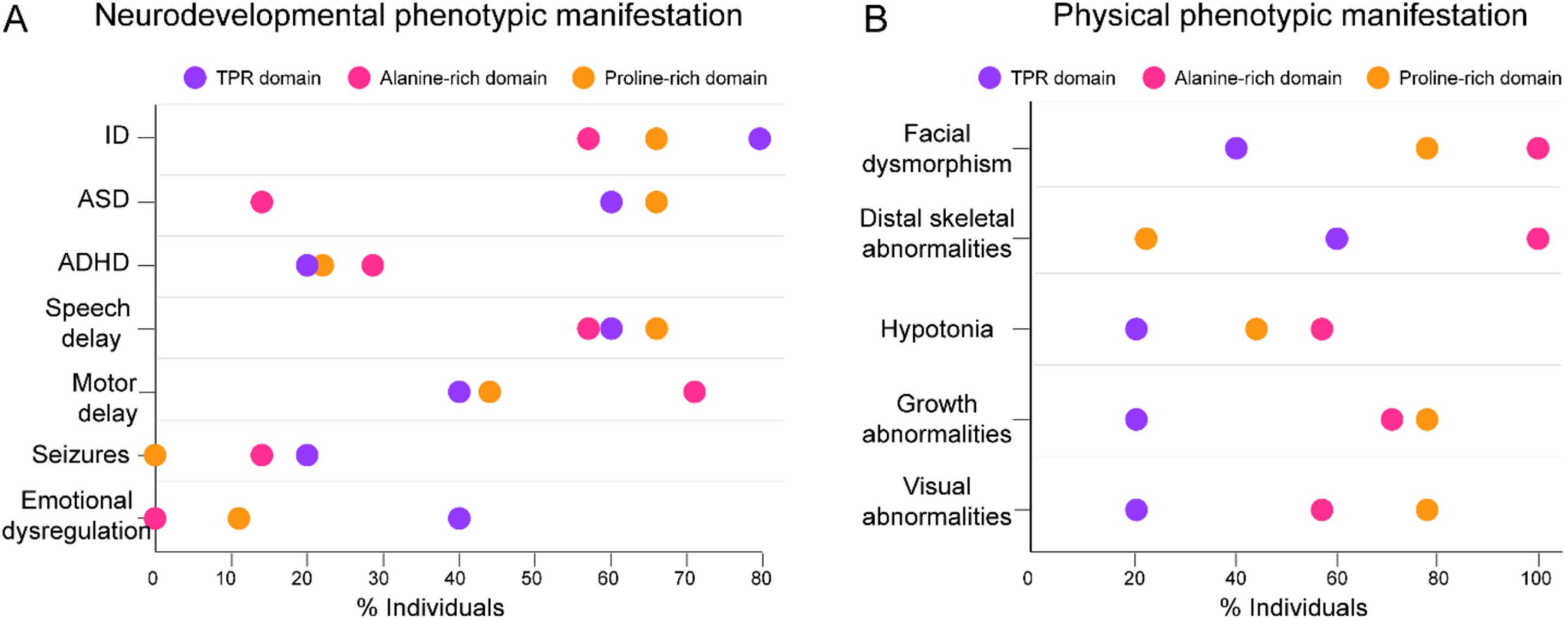
Neurodevelopmental and physical phenotypic manifestations in individuals with variants affecting TPR, Alanine-rich, and Proline-rich domains. **(A)** Percentage of individuals with variants in the TPR (purple), Alanine-rich (pink), and Proline-rich (orange) domains associated with different neurodevelopmental phenotypic manifestations. **(B)** Percentage of individuals with variants in the TPR (purple), Alanine-rich (pink), and Proline-rich (orange) domains associated with different physical phenotypic manifestations. Total number of cases analyzed in this subset (n = 21). Cases with variants in TPR (n = 5), Alanine-rich (7), and Proline-rich (n = 9) domains.

From this group of studies reporting ZMIZ1 variants associated with developmental and neurodevelopmental disorders (36 cases), we selected those that included in-depth neurological and physical phenotypical descriptions of individuals for further analysis (21 cases). In this subset, 5 cases correspond to variants in the TPR domain, 7 cases correspond to variants in the Alanine-rich domain, and 9 cases correspond to variants in the Proline-rich domain. We calculated the percentage of individuals with variants in the TPR, Alanine-rich, or Proline-rich domains with a diagnosis of ID, ASD, ADHD, speech delay, motor delay, seizures, and emotional dysregulation (Figure 3). In this subsample of individuals, we confirmed that the percentage of cases with variants in the Alanine-rich domain who display ASD is low (14%) compared to the percent of individuals with variants in TPR and Proline-rich diagnosed with ASD (60 and 66%, respectively) (Figure 3A). The percentage of individuals with variants associated with ID and speech delay was high in all domains (66% ID and speech delay for variants in Proline-rich, 80% ID and 66% speech delay for variants in TPR, and 57% ID and speech delay for variants in Alanine-rich). ADHD and the presence of seizures are phenotypes with weaker association with variants in these domains (less than 30% of individuals). Seventy-one percent of individuals with variants in Alanine-rich presented motor delay, compared to 44% in Proline-rich and 40% in TPR domains. However, zero individuals with variants in Alanine-rich presented emotional dysregulation, while 40% of individuals with variants in TPR presented emotional dysregulation (Figure 3A). Other phenotypes of potential neurodevelopmental origin, such as hearing loss and abnormal sleep, were reported in this subset of patients with variants located across ZMIZ1 with no association to a specific domain. Thirty-three percent of patients presented hearing loss and 14% presented sleep abnormalities.

In this subset of 21 cases, we also calculated the percentage of individuals with variants in the TPR, Alanine-rich, or Proline-rich domains with a diagnosis of physical abnormalities such as facial dysmorphism, distal skeletal abnormalities (in hands and feet), hypotonia, growth abnormalities such as growth delay, and visual abnormalities such as strabismus and amblyopia (Figure 3B). Overall, we observed that physical abnormalities are more prevalent in individuals with variants in the Alanine-rich domain than in other domains. Of the individuals with variants affecting the Alanine-rich domain, 100% presented facial dysmorphism, 100% presented distal skeletal abnormalities, 71% presented growth abnormalities, and 57% presented hypotonia and visual abnormalities (Figure 3B). However, of the individuals with variants affecting the TPR domain, only 40% presented facial dysmorphism, 60% presented distal skeletal abnormalities, and only 20% presented hypotonia, visual abnormalities, or other growth abnormalities (Figure 3B). For individuals with variants affecting the Proline-rich domains, the most prevalent physical phenotypic manifestations are facial dysmorphism, visual abnormalities, and other growth abnormalities, with 78% of individuals with Proline-rich variants presenting these phenotypes (Figure 3B).

In summary, ID, speech delay, motor delay, and facial dysmorphism are the most frequently observed neurodevelopmental and physical phenotypic manifestations in patients with ZMIZ1 mutations, regardless of the protein domain affected by the variants. However, SNVs in the TPR domain are more frequently associated with ASD and emotional dysregulation diagnosis, and not strongly related to physical phenotypic manifestations. In contrast, variants in the Alanine-rich domain are frequently associated with motor delay diagnosis and are concomitant with the presence of physical phenotypic manifestations.

### Association between ZMIZ1 variants, domains, and brain morphological abnormalities

We next examined the ratio of subjects with SNVs showing morphological abnormalities in the brain and cranium. Individuals were evaluated via MRI, CT scan, or cranial measurements. Most individuals presented morphological abnormalities. Seventy-eight percent of individuals assessed by MRI, CT scan, or cranial measurements presented abnormalities in the brain and/or cranial size or shape. When analyzing individuals assessed exclusively via MRI, 71% presented brain morphological abnormalities.

Since examination via MRI allows for a detailed examination of brain structures, we further classified the morphological brain abnormalities reported into specific categories and analyzed their ratio in MRI-examined individuals (Table 1). The most common morphological abnormality was microcephaly (64%), in some cases reported concomitantly with skull shape anomalies such as dolichocephaly or brachycephaly (Table 1). Fifty-seven percent of MRI-examined individuals presented abnormalities in the cortex, such as heterotopias in the cortical white matter and near the ventricles, or abnormalities in septum pelucidum and ventricles. Abnormalities in the cortex and cortical white matter, including thinner cortex, thinner white matter, or abnormal cortical gyra, were observed in 50% of MRI-examined individuals (Table 1). Abnormalities in the corpus callosum, with or without heterotopias near the ventricular space, were observed in 21% of individuals. Alterations in the meningeal space and cerebellar atrophy were less frequently reported (14 and 7%, respectively) (Table 1).

**Table 1:** Morphological abnormalities evaluated via MRI in subjects carriers of ZMIZ1 SNVs.

Abnormalities	% Evaluated by MRI
Microcephaly and/or Skull shape	64% (9/14)
Cortex, midline, Ventricles	57% (8/14)
Cortex and white matter	50% (7/14)
Corpus callosum and heterotopies	21 (3/14)
Meningeal space	14% (2/14)
Cerebellar atrophy	7% (1/14)

**Figure 4:**
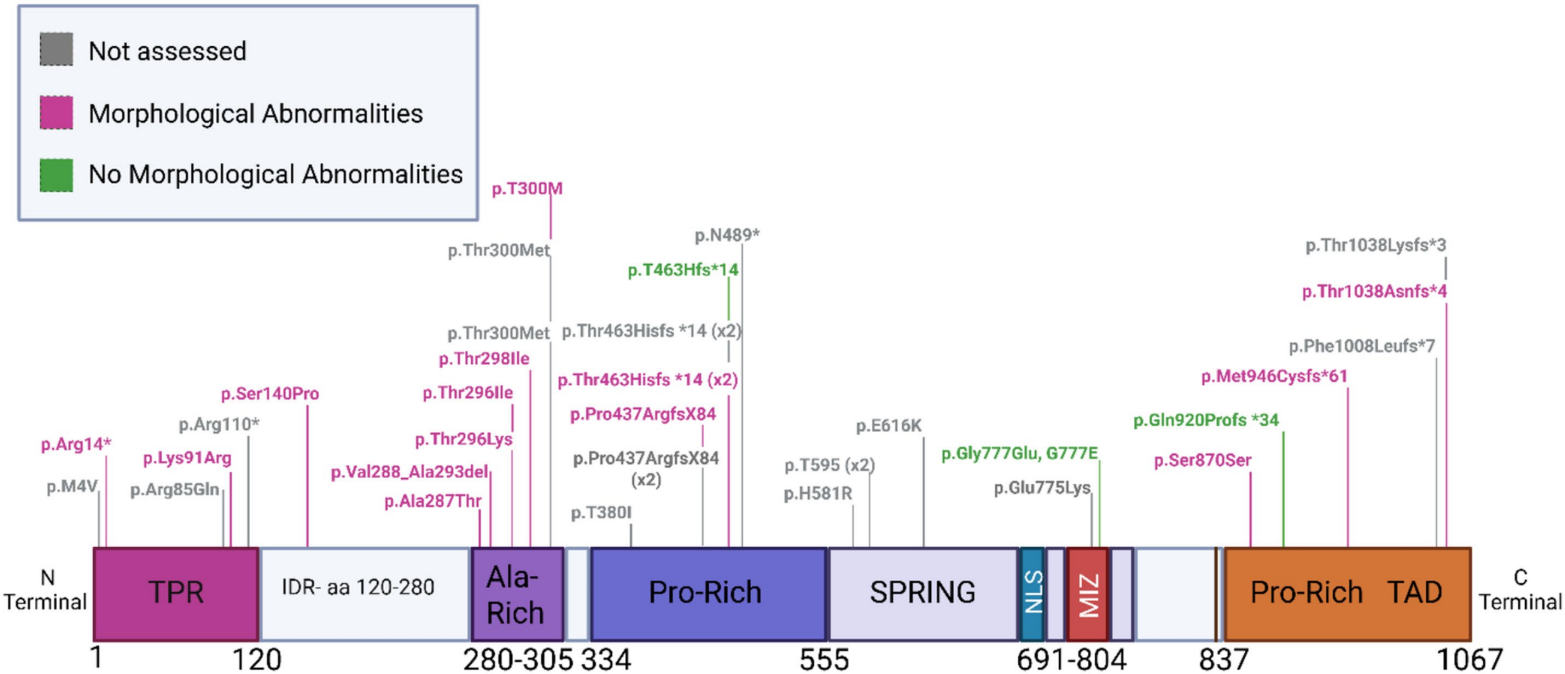
ZMIZ1 map illustrating the location of de novo SNVs identified ZMIZ1 carriers presenting morphological abnormalities in the cranium and/or brain. ZMIZ1 protein map illustrating the location of *de novo* SNVs identified in ZMIZ1 carriers presenting morphological abnormalities (magenta) in the cranium and/or brain. Variants in individuals without morphological abnormalities in the brain and/or cranium (green). Variants in individuals not assessed for brain and/or cranial abnormalities (grey).

Next, we examined whether the presence of variants across different ZMIZ1 domains is related to the presence of morphological abnormalities (Figure 4). Of the 5 individuals carrying variants in the TPR domain, two underwent morphological assessment and both presented morphological abnormalities (2/2 individuals, 100% affected) (Figure 4).

Only one SNV in the IDR aa 120–280 region of the ZMIZ1 sequence was found among all reported cases. This individual presented morphological abnormalities in the brain (1/1 individual, 100% affected; Figure 4). Of the 8 individuals presenting variants in the Alanine-rich domain, 6 were evaluated for morphological abnormalities, and all presented alterations (6/6 individuals, 100% affected; Figure 4). Of the 10 individuals presenting variants in the central Proline-rich domain, 4 were evaluated, and 3 presented alterations (3/4 individuals, 75% affected; Figure 4). Similarly, 4 individuals presenting variants in the C-terminal Proline-rich TAD domain were evaluated, and 3 presented morphological alterations (3/4 individuals, 75% affected; Figure 4). In contrast, of the 6 individuals presenting variants in the SPRING-MIZ domain, only one was evaluated. This individual did not show brain alterations (0/1 individual, 0% affected; Figure 4).

In summary, morphological alterations in the brain and cranium are highly prevalent in individuals with SNVs in the ZMIZ1 coding sequence. Brain and cranial alterations have been observed in individuals with mutations across all ZMIZ1 protein domains, and mutations in specific domains are not associated with a higher incidence of brain morphological alterations. While a slightly higher frequency of morphological abnormalities was associated with variants occurring in N-terminal regions (TPR through Alanine-rich domain), the number of assessed individuals is too low to attempt any other analysis. A greater sample of individuals with ZMIZ1 variants assessed for brain morphological alterations will be needed to establish any domain-specific association.

### Modeling missense mutations in Alanine-rich and TPR domain

**Figure 5:**
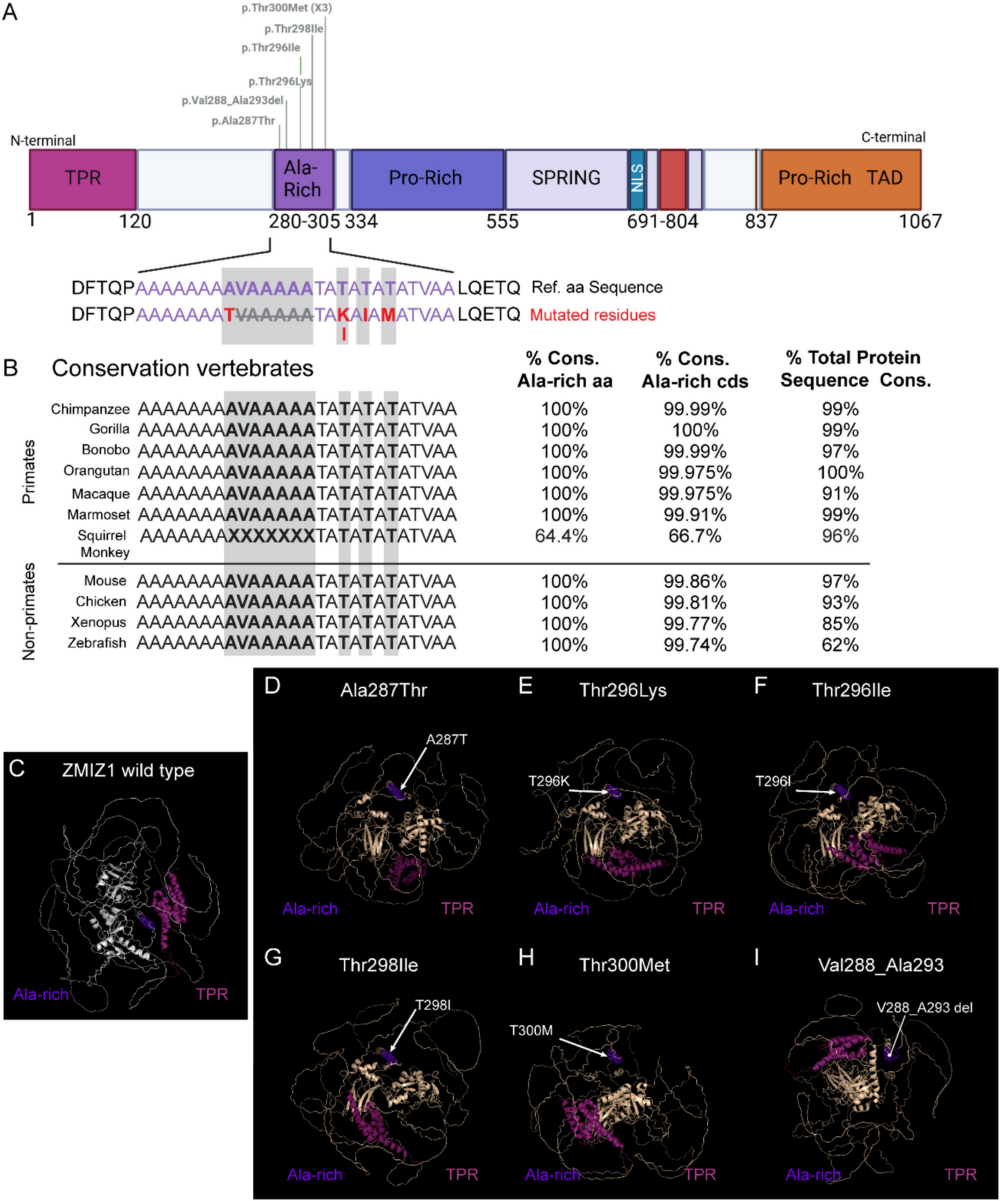
Modeling of missense variants in the Alanine-rich domain and conservation of residues across species. **(A)** ZMIZ1 map illustrating the location of SNVs in the Alanine-rich analyzed. **(B)** Alignment of the amino acid sequence in the Alanine-rich domain (purple) across primates and other vertebrate species, highlighting the mutated residues in the human cases (red and grey shading). **(C–I)** Predicted 3D structural models of human wild-type ZMIZ1 **(C)**, and ZMIZ1 with mutations in the Alanine-rich domain **(D–I)**. The Alanine-rich domain (purple) is aligned in all the models and serves as a fixed point to center the structure. TPR domain (magenta) has been colored to compare the relative position of these domains in the structure. Arrows indicate the location of mutated residues in the model.

The Alanine-rich domain has the highest accumulation of disease-causing SNVs. Our phenotypic analysis supports a strong association of variants in this domain with the diagnosis of ID, motor delay, facial dysmorphisms, distal skeletal abnormalities, and brain/skull morphological alterations. This suggests a low tolerance to variations in this domain, and that even changes in a single amino acid may significantly affect ZMIZ1 structure and function. To investigate this hypothesis we performed 3D modeling and prediction analysis of SNVs reported in the Alanine-rich domain (Figure 5). The Alanine-rich domain amino acid sequence, including the residues affected by the missense variants identified in NDD patients, is highly conserved across vertebrate species from zebrafish to humans (Figure 5B). All primate species analyzed, except for the squirrel monkey (*Saimiri boliviensis*), showed 100% amino acid conservation, and conservation of the Alanine-rich coding sequence between 99.91 and 100% (Figure 5B). We performed 3D modeling and prediction analysis of ZMIZ1 with the missense variants in the Alanine-rich domain reported in patients and compared them with the ZMIZ1 reference structure (Figures 5C–I). Comparison with the wild-type reference structure reveals alterations in the overall configuration of ZMIZ1 carrying single amino acid missense mutations (Ala287Thr, Thr296Lys, Thr298Ile, Thr298Ile, Thr300Met) or deletion Val288-Ala293 in Alanine-rich domain. Mutations change the relative position of the Alanine-rich domain in the protein model, displacing it away from the inner core of the protein (Figures 5D–I).

**Figure 6:**
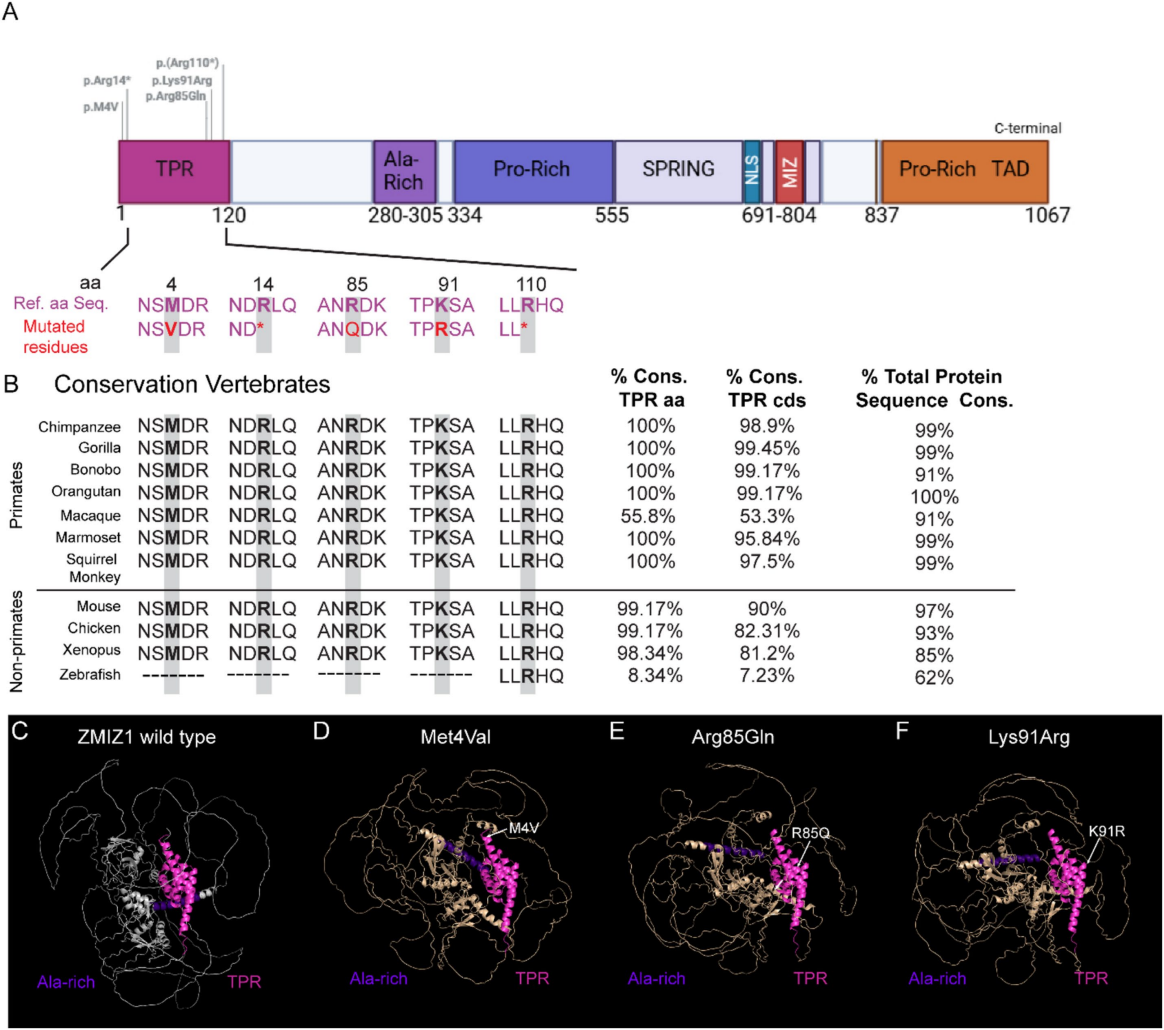
Modeling of missense variants in the TPR domain and conservation of residues across species. **(A)** ZMIZ1 map illustrating the location of SNVs in the TPR domain. **(B)** Alignment of the amino acid sequence in the TPR domain across primates and other vertebrate species. Only residues mutated in the human cases (red and grey shading) and flanking amino acids are shown. The position of the mutated residue in the TPR domain is indicated with a number. Asterisks denote variants resulting in termination of the protein sequence. **(C–F)** Predicted 3D structural models of human wild-type ZMIZ1 **(C)**, and ZMIZ1 with mutations in the TPR domain **(D–F)**. The TPR domain (magenta) is aligned in all the models and serves as a fixed point to center the structure. Alanine-rich domain (purple) has been colored to compare the relative position of these domains in the structure. Arrows indicate the location of mutated residues in the models.

Since SNVs in the TPR domain have not been previously associated with neurological phenotypes in the ZMIZ1 neurodevelopment syndrome, we performed evolutionary conservation analysis of the TPR amino acids and coding sequence, and 3D modeling of SNVs resulting in single amino acid missense mutations in the TPR (Met4Val, Arg85Gln, Lys91Arg) (Figure 6). The amino acid sequence corresponding to the TPR domain, including the residues affected by the missense variants identified in NDD patients, is conserved across all primate species analyzed, except for the macaque (55.8% conservation, Figure 6B), which has a longer coding sequence for the TPR domain. The TPR amino acid sequence in macaques is longer than in humans and other primates, containing 35 additional residues before the first amino acid in the TPR in other primates. Although the presence of this additional upstream sequence in macaques decreases the overall percentage of conservation of the TPR, the sequence of the TPR overlapping with the sequence in humans and other primates is identical. The percentage of conservation of the TPR domain in other vertebrates such as mice, chicken, and xenopus, is also high (98.34 to 99.17%; Figure 6B). However, the TPR domain coding sequence and amino acid sequence of zebrafish are poorly conserved (8.34% amino acid conservation). We performed 3D modeling and prediction analysis of ZMIZ1 with three missense variants resulting in single amino acid substitution in the TPR and compared them with the ZMIZ1 structure (Met4Val, Arg85Gln, Lys91Arg; Figures 6C–F). Comparison with the wild-type reference structure reveals alterations in the configuration of ZMIZ1 protein. Prediction models suggest that these mutations do not significantly change the structure of the TPR domain, however, they alter the relative positions of the TPR to the Alanine-rich domain (Figures 6C–F), and likely to other domains.

## Discussion

Understanding the effect on the pathogenicity of various mutation types in different domains within the ZMIZ1 protein is crucial for the improvement of diagnostic measures, as well as the resulting prescription of necessary therapies and treatments. Establishing a solid connection between mutation location and the expected neuropathological dysfunction will be paramount in the early prediction of a child’s future NDD condition. The ability to diagnose an NDD, such as ID and ASD, using genetic testing at early stages of development has the potential to provide early intervention when the brain is at its most plastic state, and therefore to make improvements in the overall quality of life of the child and their family members. Our analysis provides new insight into the differential impact of ZMIZ1 variants in neurodevelopment and supports associations between SNVs, missense mutations, affected protein domains, and neurological phenotypes based on the variants reported in the literature.

The pioneering study by Carapito and colleagues in 2019 (Carapito et al., 2019) provided the first comprehensive description of a cohort of patients with variants in ZMIZ1, establishing that alterations in this gene cause a syndromic neuro-developmental disorder. Carapito et al. already reported a strong association between ID and developmental delay, observed in every patient in the study, however, a significant degree of heterogeneity in the phenotypes and comorbidities was evident in this first cohort of patients. Over the years, multiple studies have reported individuals with neurodevelopmental disorders carrying ZMIZ1 variants (Latchman et al., 2020; Córdova-Fletes et al., 2015; Lu et al., 2022; Phetthong et al., 2021; He et al., 2024; Sheth et al., 2023; Alqahtani et al., 2023; Zhou et al., 2022; Deng et al., 2022; Valind et al., 2021; Bartolomaeus et al., 2023; Brea-Fernández et al., 2022; Schluth-Bolard et al., 2019). This has provided a larger sample of cases and variants, and the opportunity to compare the effect of mutations affecting different parts of the protein. Interestingly, variants affecting non-coding and regulatory regions of ZMIZ1 have been also reported in patients with NDDs (Liu et al., 2018; Ben Khalaf et al., 2016). This indicates that variants affecting the regulation of ZMIZ1 expression are also involved in NDDs, highlighting its functional importance in neurodevelopment. Our study focused on comparing variants across the ZMIZ1 coding sequence, and their impact on neurodevelopmental phenotypes.

Interestingly, we find a strong association between ID, motor delay, facial dysmorphism, and distal skeletal abnormalities with variants in the Alanine-rich domain. This domain has been previously reported to accumulate the highest number of disease-causing variants (Rajan et al., 2024). Notably, we also find a significant association between variants in the TPR domain and diagnosis of ASD, with or without ID, and altered emotional regulation, but no strong association with facial or other growth abnormalities. The TPR domain enables the interaction of ZMIZ1 with NOTCH1 (Rakowski et al., 2013; Wang et al., 2018; Pinnell et al., 2015) but has not been previously directly related to NDDs.

Our prediction models of ZMIZ1 with mutations in the Alanine-rich and TPR domains suggest potential structural changes that may underlie protein dysfunction. Interestingly, the effect of mutations Lys91Arg, in the TPR domain, and Thr300Met, in the Alanine-rich domain, have been experimentally tested in mouse cortical neurons. Expression of these mutant proteins altered neuron migration, differentiation, and neurite growth in mouse cortical neurons (Carapito et al., 2019). The effects of these mutations on cell migration may be mediated via the interaction of ZMIZ1 with NOTCH1, which increases the activity of NOTCH1 (Rakowski et al., 2013; Wang et al., 2018; Pinnell et al., 2015). NOTCH1 controls the activity of Rac1 (Bircher and Koleske, 2021), a well-known cytoskeletal regulator essential for neuron migration and whose dysregulation is known to cause heterotopias (Chen et al., 2023; Kawauchi, 2003; Konno et al., 2005). Importantly, Rac1 regulates brain and craniofacial development (Bircher and Koleske, 2021), and has been involved in several NDDs (Gahankari et al., 2021). This suggests a potential pathway dysregulated by these ZMIZ1 mutations that may underlie some of the phenotypes observed in the patients. Moreover, the effects of these mutations on the differentiation and growth of mouse cortical neurons might be mediated via the interaction of ZMIZ1 with androgen receptor (AR), which is a critical regulator of neural progenitor proliferation, neuron differentiation (La Rosa et al., 2021), and connectivity (Karliner et al., 2024). Dysregulation of AR pathway may result in microcephaly or atrophy in the cerebral and cerebellar cortex, which have been observed in some human patients affected by ZMIZ1 syndrome. Overall, our models suggest pathogenic effects of mutations in the TPR and Alanine-rich domains of ZMIZ1, leading to dysregulation of signaling pathways known to produce phenotypes consistent with those observed in the human ZMIZ1 syndrome.

Recent loss-of-ZMIZ1-function studies in mice showed abnormalities in specific neuron populations in the cerebral cortex (Rajan et al., 2024; Rajan et al., 2024). Callosal projection neurons (CPN) in the superficial cortical layers and the corticothalamic neurons (CTN) in the deep layers were affected by ZMIZ1 loss of function (Rajan et al., 2024). These neuron types are affected in several NDDs (Rubenstein, 2011), and their abnormal development produces alterations consistent with some of the phenotypes observed in patients with ZMIZ1 mutations. CPN’s axons form the corpus callosum, connect the brain hemispheres, and are essential for high-level cognitive functions (Fame et al., 2011; Fenlon and Richards, 2015; De León Reyes et al., 2019; Hinkley et al., 2012). Disruption of CPN or corpus callosum development is a common feature in NDD disorders such as ASD and ID and leads to impaired emotional regulation, social interaction, communication, and cognitive functions (Hinkley et al., 2012; Paul et al., 2007; Paul et al., 2014). These neurological phenotypes have been observed in the patients carrying ZMIZ1 variants analyzed in this study. CTN are affected in ZMIZ1 mutant mice and are important to sensory processing, cognitive processing, memory, and regulation of sleep cortical activity (Briggs and Usrey, 2008; Murphy and Briggs, 2023; Galazo et al., 2016; Feliciano-Ramos et al., 2023). Phenotypes observed in patients with ZMIZ1 mutations, such as hearing loss, cognitive and learning disability, and sleep alterations, may be related to dysregulation of CTN functions.

In summary, our study supports the differential impact of mutations across ZMIZ1 domains and their association with distinct neurodevelopmental phenotypes in individuals with ZMIZ1 variants. Mutations in TPR and Alanine-rich domains are predicted to cause alterations of ZMIZ1 structure and functions that lead to neurological phenotypes consistent with those observed in the human ZMIZ1 neurodevelopmental syndrome.

## Materials and methods

### Study population

Analysis performed using de-identified data from 15 publicly available studies describing mutations in ZMIZ1 over the past 10 years.

Inclusion criteria: we included a total of 36 affected individuals with a diagnosed NDD and harboring SNVs or deletions affecting ZMIZ1 coding region. We excluded any individuals with mutations in ZMIZ1 that are classified as translocations, inversions, or variants in ZMIZ1 splice donors/acceptors.

Variants description: Most SNVs were *de novo* variants. Of these 36 ZMIZ1 mutations, 35 were SNV: 5 located in the TPR domain, 7 in the Alanine-rich domain, 10 in the Central Proline-rich domain, 6 in the Spring-MIZ region, 6 in the Pro-rich/TAD domain, and 1 in the Intrinsically disordered region (IDR) between the TPR and the Alanine-rich domains (aa 120–288). Of the 35 SNVs, 2 are synonymous mutations and 33 produce missense mutations. In addition, one deletion located in the Alanine-rich domain was included (Supplementary Table 1). Due to the difficulties in phenotypic interpretation, we did not include individuals with copy number variants encompassing genes other than ZMIZ1. The locations of the variants were mapped within the ZMIZ1 reference sequence and protein domains. Mutations in splice donors/acceptors or causing translocations and gene fusions were not included in this study.

### Variants pathogenicity scores

Pathogenicity scores were obtained from AlphaMissense or PolyPhen. AlphaMissense categorises ‘missense’ mutations as either ‘likely pathogenic’, ‘likely benign’, or ‘uncertain’, producing a score that estimates the likelihood of a variant being pathogenic. Missense variants with scores 0.0–0.34 are classified as “benign,” missense variants with scores 0.34–0.564 are classified as “un-certain,” and variants with scores 0.564–1.0 are classified as “pathogenic” (Cheng et al., 2023). AlphaMissense scores for the 33 missense variants included in this study are listed in Supplementary Table 1. AlphaMissense scores for all possible missense variants in ZMIZ1 can be accessed at https://alphafold.ebi.ac.uk/entry/Q9ULJ6.The pathogenicity heatmap is generated with AlphaMissense AI model resource publicly available at the AlphaFold Protein Structure Database developed by Google DeepMind and EMBL-EBI.

The AlphaMissense pathogenicity heatmap contains the scores estimating the likelihood of pathogenicity and classifications for each possible amino acid substitution within the human ZMIZ1 protein. The heatmap is a resource publicly available at the AlphaFold Protein Structure Database developed by Google DeepMind and EMBL-EBI. It is accessible and searchable at https://alphafold.ebi.ac.uk/entry/Q9ULJ6.

PolyPhen scores were directly extracted from Ensembl database using the full variant list for Zmiz1: transcript ID ENST00000334512.10 ZMIZ1-201, available at: https://useast.ensembl.org/Homo_sapiens/Transcript/Variation_Transcript/Table?db=core;g=ENSG00000108175;r=10:7906896679316519;t=ENST00000334512;v=rs746314274;vdb=variation;vf=659724931.

For those variants not listed in the Ensembl variants list, scores were generated using the PolyPhen-2 prediction tool.^1^ For scores using the PolyPhen-2 tool, searching parameters introduced for variants were protein name (ZMIZ1), amino acid position, amino acid symbol substituted for a given position, and resulting amino acid specified in the variant description. The resulting scores from HumDiv model are reported since HumDiv is trained on human variation data and is better for evaluating rare alleles potentially involved in complex traits (Adzhubei et al., 2010).

ClinVar accession numbers were included for those variants in ClinVar. When multiple pathogenicity records were associated with a variant, the most recent one was reported. All ClinVar records for ZMIZ1 variants are accessible at: https://www.ncbi.nlm.nih.gov/clinvar?term=%22ZMIZ1%22%5BGENE%5D&cmd=DetailsSearch&log$=activity.

### 3D protein structure prediction and conservation analysis

Protein domain and protein folding predictions were performed using the machine-learning protein structure prediction model AlphaFold2.^2^ The ZMIZ1 reference protein sequence and structures were retrieved from the AlphaFold Protein Structure Database (AlphaFold DB) (Tunyasuvunakool et al., 2021) by ZMIZ1 UniProt accession code (Q9ULJ6). The predicted protein structures resulting from single nucleotide variants in ZMIZ1 affecting the TPR and Alanine-Rich domains were modeled by the standalone version of AlphaFold (Jumper et al., 2021) using the FASTA sequence. The per-residue local distance difference test (pLDDT) confidence scores for the protein prediction models were retrieved from the AlphaFold DataBase as in (Pak et al., 2023). Only the top-ranked predicted structures were analyzed. Alignment and visualization of 3D structures were performed using Chimera (Pettersen et al., 2004) and PyMOL as previously described (Booth et al., 2024). Prediction model comparisons and alignment of prediction models with mutated amino acids in the Alanine-rich domain or in the TPR domain were performed using PyMOL automated multi-step superposition algorithm ‘align’ tool, fixing the mutated domain as the origin for comparison with the reference structure. For protein conservation analysis, sequences from multiple species were compared to the human ZMIZ1 sequence using the genome alignment tool of ECR^3^ and the gene orthologue alignment tool of the Ensembl database.^4^

## Data Availability

The original contributions presented in the study are included in the article/supplementary material, further inquiries can be directed to the corresponding author.

## Footnotes

^1^ http://genetics.bwh.harvard.edu/pph2/index.shtml

^2^ https://neurosnap.ai/services

^3^ www.ecrbrowser.dcode.org

^4^ www.ensembl.org
