# Supplementary material for: Association of genetic variants, protein domains, and phenotypes in the ZMIZ1 syndromic neurodevelopmental disorder
Source: Front Neurosci. 2025 Jun 3;19:1605762. doi: 10.3389/fnins.2025.1605762 (PMC12170594; doi:10.3389/fnins.2025.1605762)
Supplement: Supplementary file 1 [file Table_1.docx]

**Supplemental Material**

| **Protein Domain** | **Variant** | **Source/Study** | **AlphaMissense** | **PolyPhen** | **ClinVar** |
| --- | --- | --- | --- | --- | --- |
| **TPR** | p.M4V  rs1414523764 | Zhou et al., 2022 | 0.273 | 0.945 | / |
|  | p.Arg14* NP_065071.1:p.Arg14Ter | Deng et al., 2022 | / | / | VCV003819528.1/ Pathogenic |
|  | p.Arg85Gln  rs762370940 | Sheth et al., 2023 | 0.971 | 0.883 | / |
|  | p.Lys91Arg  rs1554817910 | Carapito et al., 2019 | 0.170 | 0.903 | VCV000487354.5/ Pathogenic/  Likely pathogenic |
|  | p.Arg110*  NP_065071.1:p.Arg110Ter | Alqahtani et al., 2023 | / | / | VCV002503420.3/  Likely pathogenic |
| **IDR aa 120-280** | p.Ser140Pro  NP_065071.1:p.Ser140Pro | Bartolomaeus et al., 2023 | 0.092 | 0.098 | VCV000995992.2/  Uncertain significance |
| **IDR Alanine-Rich** | p.Val288_Ala293del  rs751961170 | He et al., 2024 | / | / | VCV003370510.1/  Likely pathogenic |
|  | p.Ala287Thr  rs1472883107 | Carapito et al., 2019 | 0.698 | 0.978 | VCV000996609.2/  Uncertain significance |
|  | p.Thr296Lys  NP_065071.1:p.Thr296Lys | Carapito et al., 2019 | 0.997 | 0.92 | VCV000694591.4/  Pathogenic |
|  | p.Thr296Ile  NP_065071.1:p.Thr296Ile | Carapito et al., 2019 | 0.999 | 0.97 | VCV000996613.3/  Pathogenic |
|  | p.Thr298Ile  rs1853549548 | Carapito et al., 2019 | 0.998 | 0.89 | VCV000996610.1/  Likely pathogenic |
|  | p.Thr300Met  rs1589579500 | Carapito et al., 2019 | 0.993 | 0.978 | VCV000694588.8/  Pathogenic |
|  | p.Thr300Met  rs1589579500 | Carapito et al., 2019 | 0.993 | 0.978 | VCV000694588.8/  Pathogenic |
|  | p.Thr300Met  rs1589579500 | Zhou et al., 2022 | 0.993 | 0.978 | VCV000694588.8/  Pathogenic |
| **IDR Proline-Rich** | p.T380Ile | Zhou et al., 2022 | 0.178 | / | / |
|  | p.Thr463Hisfs*14  NP_065071.1:p.Thr463fs | Carapito et al., 2019 | 0.285 | 0.865 | VCV000694593.4/  Pathogenic |
|  | p.Pro437ArgfsX84  rs138149224 | Latchman et al., 2019 | 0.498 | 0.047 | / |
|  | p.Pro437ArgfsX84  rs138149224 | Latchman et al., 2019 | 0.498 | 0.047 | / |
|  | p.Pro437ArgfsX84  rs138149224 | Latchman et al., 2019 | 0.498 | 0.047 | / |
|  | p.Thr463Hisfs*14  NP_065071.1:p.Thr463fs | Carapito et al., 2019 | 0.285 | 0.865 | VCV000694593.4/  Pathogenic |
|  | p.Thr463Hisfs*14  NP_065071.1:p.Thr463fs | Carapito et al., 2019 | 0.285 | 0.865 | VCV000694593.4/  Pathogenic |
|  | p.Thr463Hisfs*14  NP_065071.1:p.Thr463fs | Carapito et al., 2019 | 0.285 | 0.865 | VCV000694593.4/  Pathogenic |
|  | p.Asn489* | Zhou et al., 2022 | / | / | Likely benign |
|  | p.Thr463Hisfs*14  NP_065071.1:p.Thr463fs | Zhou et al., 2022 | 0.285 | 0.865 | VCV000694593.4/  Pathogenic |
| **SPRING-MIZ** | p.His581Arg | Zhou et al., 2022 | 1 | 0.999 | / |
|  | p.Thr595=  NC_000010.11:g.79299168G>A | Zhou et al., 2022 | / | / | VCV002104953.3/  Benign |
|  | p.Thr595Met  rs746314274 | Zhou et al., 2022 | 0.250 | 0.508 | VCV002175369.4/  Uncertain/Likely benign |
|  | p.Glu616Lys | Zhou et al., 2022 | 1 | 0.913 | / |
|  | p.Glu775Lys  rs759993930 | Sheth et al., 2023 | 0.998 | 0.927 | / |
|  | p.Gly777Glu,G777E | Lu et al., 2022 | 0.999 | 0.913 | / |
| **IDR Proline-Rich TAD** | p.Ser870Ser  NP_065071.1:p.Ser870= | Carapito et al., 2019 | / | / | VCV000996611.1  Likely pathogenic |
|  | p.Gln920Profs*34  rs1462821614 | Carapito et al., 2019 | 0.053 | 0.992 | / |
|  | p.Met946Cysfs*61  NP_065071.1:p.Met946fs | Carapito et al., 2019 | 0.599 | 0.707 | VCV000996614.1,  Likely pathogenic |
|  | p.Phe1008Leufs*7  NP_065071.1:p.Phe1008fs | Carapito et al., 2019 | 0.963 | 0.995 | VCV000996612.1/  Likely pathogenic |
|  | p.Thr1038Asnfs*4 | Carapito et al., 2019 | 0.290 | 0.969 | VCV003336982.2/  Pathogenic |
|  | p.Thr1038Lysfs*3 | Valind et al., 2021 | 0.782 | 0.920 | RCV000856609.3/  Pathogenic |

**Table S1.** Summary of SNVs reported in individuals with developmental and NDDs. Location in ZMIZ1 protein domain, predicted pathogenicity scores by AlphaMissense, PolyPhen, and ClinVar. AlphaMissense score estimates the likelihood of a variant being pathogenic (“benign” scores 0.0 - 0.34; “uncertain” scores 0.34 - 0.564; “pathogenic” scores 0.564 - 1.0). PolyPhen score ranges from 0.0 (benign) to 1.0 (deleterious). ClinVar records obtained from https://www.ncbi.nlm.nih.gov/clinvar?term=%22ZMIZ1%22%5BGENE%5D&cmd=DetailsSearch&log$=activity
